# Emphasizing the Clinical Diagnosis of Acute Appendicitis Amidst Technological Advancements

**DOI:** 10.7759/cureus.60555

**Published:** 2024-05-18

**Authors:** Katie Wang, Munyaradzi G Nyandoro, Mary Teoh, Richard Naunton-Morgan

**Affiliations:** 1 General Surgery, Sir Charles Gairdner Hospital, Perth, AUS; 2 General and Colorectal Surgery, Sir Charles Gairdner Hospital, Perth, AUS

**Keywords:** ultrasound scan, contrast-enhanced computed tomography, negative appendicectomy, radiological imaging, clinical diagnosis, appendicitis

## Abstract

Background

The clinical diagnosis of acute appendicitis (AA) can be challenging. This study aimed to evaluate the significance of this diagnosis amidst technological progress. It compared clinical diagnosis to radiology-aided diagnostic outcomes and negative appendicectomy rates (NAR).

Methodology

This study conducted a single-center retrospective and prospective cohort observational study on all adult patients presenting with suspected AA in 2018 at a major tertiary teaching hospital in Perth, Western Australia. Key demographics, clinicopathological, radiology, and operative reports were reviewed. Data were analyzed using SPSS v.27.

Results

Of 418 patients with suspected AA, 234 (56%) were in the retrospective group. The median age was 35 (IQR=26), and 224 (54%) were female. The overall NAR was 18.6% (95% CI (14.8-22.4)) and 20.8% for clinical diagnosis. Notably, the NAR for ultrasound (USS)-reported AA (false positive) was 17.6% (95% CI (10.6-27.4)). Three-quarters of the patients, 298 (71.3%), had radiological imaging. The most common modality was CT 176 (59.1%), and 33 (7.9%) had both CT and USS imaging performed.

Compared with final histopathology, no significant difference was found in the accuracy of clinically diagnosed and USS-diagnosed cases, with rates of 83.5% and 82.5%, respectively (p=0.230). CT had the best positive predictive value at 82.1%. Single-modality imaging did not cause a significant surgical delay (p=0.914), but multi-modal imaging showed a non-significant trend toward delay (p=0.065).

When surgeons assessed an appendix as normal, 54 (12.9%), the histopathological assessment revealed pathology in 28 (51.9%). The inter-observer agreement was only fair to moderate, Kappa=0.46 (95% CI (0.33-0.58); p<0.001). The intraoperative identification of a normal appendix inversely correlated to the grade of the primary surgeon, which was likely related to the number of surgical personnel in the theater (p<0.001).

Conclusion

This study showed that clinical diagnosis matches the diagnostic accuracy of imaging technologies. Utilizing diagnostic imaging methods promptly and appropriately did not lead to considerable delays in surgery. Surgeons’ capability to diagnose appendicitis during surgery is moderately accurate. Most patients underwent imaging, with CT scans being the most common. Moving forward, practitioners must minimize excessive reliance on imaging techniques as this can be resource-intensive, especially in developing countries. Future clinical practice should balance embracing technological advancements and preserving essential clinical diagnostic expertise, for medicine is both a science and an art.

## Introduction

Acute appendicitis (AA) is the most common abdominal surgical emergency [1]. The classic presentation of migratory pain to the right iliac fossa, associated with nausea, vomiting, and anorexia, occurs in less than half of the cases [2], sometimes making clinical diagnosis challenging. A negative appendicectomy (NA) occurs when a histologically normal appendix is removed. The NA rate (NAR) has been reported in the literature to be between 15% and 25%. NA is associated with increased cost, prolonged hospitalization, and morbidity [3-8]. The World Society of Emergency Surgery (WSES) 2020 updated guideline recommends judicious usage of diagnostic imaging for AA [7].

In the literature, imaging has been reported to decrease the NAR [9]. Point-of-care ultrasound (POCUS) has been recommended as the most appropriate first-line diagnostic tool in adults and children [7-11]. Contrast-enhanced computed tomography (CT) is the most frequently used imaging modality, especially in adult males. CT has the lowest NAR but is associated with exposure to ionizing radiation and contrast, is contraindicated in pregnancy, and is relatively contraindicated in young female patients [11]. Delays in obtaining imaging are associated with an increased risk of perforation and sepsis [12, 13]. The increase in costs associated with imaging was reported in an Australian study by Wang E et al. [14].

This study reports a single-center experience examining the current diagnostic journey for patients presenting with suspected AA. It compares the NAR between clinical and radiology-aided diagnoses and associated outcomes.

## Materials and methods

Patients and data collection 

A single-center observational study was conducted on all adult patients with suspected AA in Perth, Western Australia, for the 2018 calendar year. A standard data collection tool was made availaNextble in electronic and hard copy formats for the on-call admitting surgical teams to capture data prospectively (pre-intra and post-operatively) for the duration of the hospital stay, and any gaps in data were filled by the investigators (MT and MN). Retrospective and prospective cohorts were included to evaluate the observer effect. The investigators reviewed and collected the clinicopathological data on demographics, diagnostic data, radiology modality and findings, operative approach and findings, recovery and complications, and histopathology reports.

Patient eligibility 

Eligible patients included in the study were adults with suspected AA who had an appendicectomy. The study also included patients who had an appendicectomy post-failed conservative management.

Outcome measures 

The primary outcome was to assess the accuracy of clinical and radiological imaging in diagnosing AA. Clinical and radiological diagnoses were compared to the final histopathology reports. Secondary outcomes included the frequency of imaging modalities, imaging determinants, and intra-operative appendicitis diagnosis accuracy.

Definitions

Clinical diagnosis of AA was defined as a diagnostic decision based on clinical history, examination, raised inflammatory markers, and other biochemistry findings. Radiological diagnosis was made when imaging was used to aid the diagnosis. The NAR was defined as the percentage of histologically normal appendices removed in patients with suspected AA. Surgical site infection (SSI) was defined as a superficial or deep space infection with associated clinical, biochemical, microbiological, and imaging correlation. Incisional hernia was diagnosed clinically or via radiological imaging (CT or ultrasound (USS)). Postoperative ileus (POI) was defined as an interval period between surgery and recovery where there is a disturbance in GI function. Obligatory POI is sub-categorized into the early period of ileus, usually mild and self-limiting, occurring in all patients undergoing surgery. Prolonged POI (PPOI) is pathological and is characterized by persistent ileus beyond the obligatory POI period. PPOI is associated with more severe symptoms, with a consensus diagnosis at day four post-op [7]. As per the therapeutic guidelines, unless there was a documented contraindication, appropriate antibiotics were defined as Cephazolin 2g and Metronidazole 500mg, given at induction or within 60 minutes of surgical incision. The Clavien-Dindo classification (CD) was used to categorize complications. Operative time was defined as the time in minutes between the initial incision and closure of the surgical wound. Thirty-day representation and readmission rates were recorded. Overall survival was determined by the date of death recorded or the date of the last follow-up.

Statistical analysis 

Baseline characteristics were described using mean (±SD), median (interquartile range), and frequencies/percentages as appropriate. Outcomes for continuous unpaired variables were analyzed with the nonparametric Mann-Whitney U test. Dichotomous outcomes were compared between groups using χ2 or Fisher’s exact tests with no adjustment for multiple comparisons. Radiological imaging utilization was reported as a proportion and 95% confidence interval for the primary outcome. A secondary analysis of the primary outcome was performed using multivariate logistic regression to assess the contribution of confounding factors. All analyses were performed using SPSS ver 27 (IBM Corp., Armonk, NY, USA), and a two-tailed p-value of <0.05 was considered statistically significant.

Ethics

Per the National Statement on Ethical Conduct in Human Research (5.1.22), the Hospital Human Research and Ethics Committee reviewed and approved the project as an alternative pathway for negligible risk projects and quality improvement (Ref # 21673).

## Results

Demographics and clinical factors

Four hundred and thirty-one patients presented with acute abdominal pain and possible AA; 411 (95.4%) were managed surgically. Of the twenty initially managed conservatively, seven (35%) failed the conservative management and proceeded to surgery. The median age was 34 (IQR = 26), and 221 (52.9%) were female. There was no observer effect between the retrospective and prospective cohorts (p > 0.05) for all key confounding variables (Table 1).

**Table 1:** Key characteristics of the retrospective and prospective groups who had surgery. Values are the number (n) of participants and percentages (%) unless otherwise indicated. * Pearson Chi-Square analysis and Fisher’s Exact Test (for cell values <5), and * Bold denotes significance at p<0.05. ^†^ M: Mean; SD; (n) = number. ^‡^ Nonparametric Mann-Whitney U test (unadjusted p-values). ^§ ^LoHS: Length of hospital stay, from day of admission to discharge and day of surgery to discharge. ^|| ^Radiology modalities: CT - all were done as contrast-enhanced, USS: Ultrasound; MRI.

Variable (N = 418)	Retrospective n = 229	Prospective n = 189	P-value ^‡^
	n (±SD) ^†^		
Age – (years: mean ± SD) ^†^	38.9 (16.5)	37.8 (17.6)	0.268
Length of Hospital (days: M ± SD) admission ^§^	2.5 (2.0)	2.8 (3.9)	0.430
Length of Hospital (days: M ± SD) post-surgery ^§^	1.9 (1.6)	2.0 (2.1)	0.226
Time to surgery (hours)	12.6 (11.5)	12.4 (13.9)	0.650
Procedure duration (mins)	59.9 (23.9)	59.1 (26.2)	0.399
	n (%)		P-value *
Female	116 (50.7)	105 (55.6)	0.318
Male	113 (49.3)	84 (44.4)	0.318
Preoperative - Clinical diagnosis	61 (26.6)	48 (25.4)	0.774
Preoperative - Probable appendicitis	50 (21.8)	33 (17.5)	0.265
Preoperative – Radiological diagnosis	118 (51.5)	108 (57.1)	0.252
Clinically normal appendix intraoperatively	36 (15.7)	21 (11.2)	0.178
Any imaging done ^||^	163 (71.2)	135 (71.4)	0.955
Contrast-enhanced CT	103 (45.0)	73 (38.6)	0.190
USS	84 (36.7)	72 (38.1)	0.766
Multiple imaging	22 (9.6)	11 (5.8)	0.153
MRI	1 (0.4)	0 (0.0)	1.000

Imaging practice parameters 

**Figure 1:**
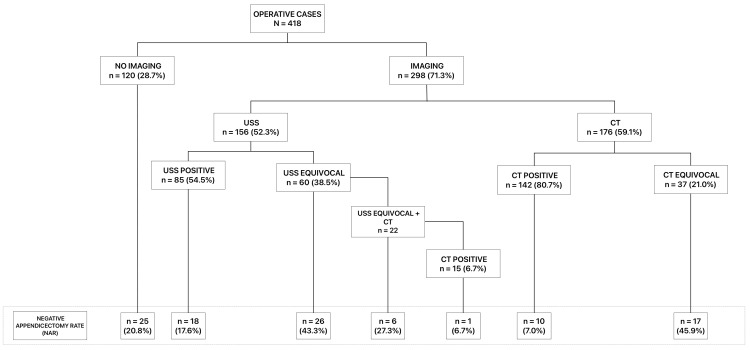
Frequency of pre-operative imaging for suspected appendicitis and corresponding negative appendicectomy rate (NAR).

Radiological imaging was used in 298 (71.3%) patients during the diagnostic work-up. Significant differences in modality were observed; CT scans were the most frequent at 176 (59.1%). The utilization rates of USS and MRI were 156 (52.3%) and 1 (0.3%), respectively (Figure 1). CT and USS were performed in 33 (7.9%) of the cases. Of the patients with an equivocal ultrasound, 23 (38.3%) subsequently had a CT. Interestingly, 10 (11.5%) of patients with a positive ultrasound for appendicitis also had a CT scan for unknown reasons. These findings on imaging modality utilization were consistent between the arms of the study, and no significant differences were observed between the retrospective and prospective groups.

Accuracy of imaging in diagnosing AA

Imaging was less predictive in patients in whom the clinical diagnosis of AA needed to be clarified. Therefore, the NAR was highest in cases where multiple imaging modalities were utilized, at 10 (30.3%). The high NAR observed in equivocal imaging for both USS and CT also supports this assertion, at 26 (43.3%) and 17 (45.9%), respectively (p<0.001). In comparison, the clinically diagnosed AA NAR was 20.8%. In cases where a positive diagnosis of AA was reported on imaging, the corresponding NAR was 17.6% for USS and 7.0% for CT (p<0.01). The accompanying positive predictive values (PPV) were 82.1%, 55.2%, and 32.8% for CT, USS, and clinical diagnosis, respectively. Likewise, the negative predictive values (NPV) were 49.9%, 48.4%, and 72.6% for CT, USS, and clinical diagnosis, respectively (Figure 1).

Determinants of imaging 

Age and gender were significantly associated with the imaging modality, with younger patients being less likely to undergo diagnostic imaging (p<0.001). Furthermore, younger patients were more likely to have USS (p<0.001), while older patients were more likely to have a CT (p<0.001). Females were more likely to undergo diagnostic imaging (p<0.001) in the form of an ultrasound (p<0.001). No statistically significant difference was observed between genders for the usage of CT (p=0.381). Patients with unclear clinical diagnoses were significantly more likely to undergo multiple imaging modalities (p<0.001) (Table 2).

**Table 2:** Determinants of imaging. Values are the number (n) of participants and percentages (%) unless otherwise indicated. ^‡ ^Nonparametric Mann-Whitney U test (unadjusted p-values). * Pearson Chi-Square analysis and Fisher’s Exact Test (for cell values <5), bolded * denotes significance at p<0.05.

Variables (N = 418)		No imaging	Imaging	No USS	USS	No CT	CT
		n (IQR)					
Age	Median (IQR)	27.0 (15)	39.5 (29)	40.0 (31)	28.0 (16)	26.0 (14)	51.0 (22)
	P-value	<0.001^‡^		<0.001^‡^		<0.001^‡^	
		n (%)					
Gender	Male n (%)	73 (80.8)	124 (41.6)	150 (57.3)	47 (30.1)	109 (45.0)	88 (50.0)
	Female n (%)	47 (39.2)	174 (58.4)	112 (42.7)	109 (69.9)	133 (55.0)	88 (50.0)
	P-value	<0.001^*^		<0.001^*^		0.381^*^	
Appendicitis severity	Normal appendix	24 (27.8)	52 (72.2)	39 (51.3)	37 (48.7)	53 (69.7)	23 (30.3)
	Early/Suppurative	86 (33.6)	170 (66.4)	167 (65.2)	89 (34.8)	165 (64.4)	91 (35.5)
	Perf/Gangrenous	9 (11.3)	71(88.8)	56 (70.0)	24 (30.0)	20 (25.0)	60 (75.0)
	P-value	<0.001^*^		0.083^*^		<0.001^*^	

Reviewing final histology findings and preoperative imaging modality reported some interesting results. Notably, among cases where histology revealed a normal appendix, 52 (72.2%) had undergone preoperative imaging. Within this subset, the NAR for CT and USS stood at 6.4% and 16.7% respectively. USS was the modality of choice in histologically normal cases or early/suppurative appendicitis (p=0.083). At the same time, CT was more frequently associated with cases likely to have either perforated or gangrenous appendicitis on final histology (p<0.001) (Table 2).

A total of 176 (59.1%) individuals underwent a CT scan, with an equal gender distribution of 88 (50%). Among males, the median age for receiving a CT scan was 48.5 (IQR 26), while for females, it was 52.0 (IQR 21), p=0.244. Conversely, a higher proportion of the 242 individuals who did not undergo a CT scan were females (55%). The median age for males who did not receive a CT scan was 28 (IQR 15), while for females, it was 25 (IQR 13), p=0.347. Despite a trend suggesting that younger females were less likely to undergo a CT scan, this observation did not reach statistical significance (Table 2).

Imaging costs and care delivery impact 

Overall, 298 (71.3%) patients received imaging before an appendicectomy. A contrast CT abdomen with portal venous phase and abdominal USS cost $980 and $250, respectively. During the study period, $210,500 was spent on imaging. The percentage of CT and ultrasound scans equivocal for appendicitis were 37 (21.0%) and 60 (38.5%), respectively. This equated to $41,160 for CT scans and $15,000 for USS across the 12 months. Single-modality imaging was not associated with a significant delay in time to surgery (p=0.914); however, multi-modal imaging showed a trend towards delay but was not statistically significant (p=0.065) (Table 3).

**Table 3:** Diagnostic imaging modality and key logistical outcomes. Values are the number (n) of participants and percentages (%) unless otherwise indicated. ^‡^ Nonparametric Mann-Whitney U test (unadjusted p-values) and * bolded denotes significance at p<0.05; ^†^ M: Mean; SD; (n): Number; ^§^ LoHS: Length of hospital stay, from day of admission to discharge and day of surgery to discharge; ^||^ Radiology modalities: CT; USS; MRI.

Variable (N = 418)	No imaging (n = 120)	Imaging (n = 298)	P-value ^‡^
Any Imaging	n (±SD) ^†^		
Time to surgery (hours)	11.2 (7.7)	13.0 (14.1)	0.914
Procedure duration (mins)	52.6 (17.0)	62.1 (27.0)	0.003
Length of hospital (days: M ± SD) admission ^§^	2.0 (1.5)	2.9 (3.4)	<0.001
USS Imaging			
Time to surgery (hours)	11.5 (9.3)	14.2 (16.7)	0.955
Procedure duration (mins)	61.0 (25.1)	56.7 (24.5)	0.057
Length of hospital (days: M ± SD) admission ^§^	2.8 (3.4)	2.5 (2.2)	0.277
CT Imaging			
Time to surgery (hours)	12.2 (12.4)	12.9 (13.0)	0.605
Procedure duration (mins)	52.2 (19.3)	69.3 (28.3)	<0.001
Length of hospital (days: M ± SD) admission ^§^	1.9 (1.3)	3.7 (4.2)	<0.001
Multi Imaging			
Time to surgery (hours)	12.0 (11.9)	18.0 (19.0)	0.065
Procedure duration (mins)	58.3 (24.2)	72.3 (30.0)	0.004
Length of hospital (days: M ± SD) admission ^§^	2.5 (2.9)	4.4 (3.6)	<0.001

Clinical accuracy of intra-operative diagnosis compared to histopathology

When surgeons assessed an appendix as normal intraoperatively, 54 (12.9%) of the histopathological assessments revealed pathology (false negative rate) in 28 (51.9%) cases. The inter-observer agreement was only fair to moderate, Kappa=0.46 [95% CI (0.33-0.58); p<0.001]. The clinical accuracy of diagnosing a normal appendix varied depending on the level of seniority. In isolation, when consultant surgeons deemed an appendix normal, pathological examination revealed pathology in 20% of these cases (i.e., 20% false negative rate). This contrasted significantly with fellows (41.2%), trainee registrars (10.0%), and service unaccredited registrars, who were likely to be heavily supervised, meaning more surgical personnel present in the OT (0.0%, p<0.001). The full spectrum of other histological findings is detailed in (Table 4).

**Table 4:** Other histopathological findings. Values are the number (n) of participants and percentages (%) unless otherwise indicated. ^†^ Calculated as a proportion of the total cohort. ^‡^ Calculated as a proportion of the total cohort.

Variables (n =74)	(n)	(%)
Inflamed appendiceal diverticulum	24	32.4
Fibrous obliteration	12	16.2
Fimbral/Ovarian cyst	11	14.9
Endometriosis	10	13.5
Sessile serrated adenoma	7	9.5
Lymphoid hyperplasia	4	5.4
Enterobius vermicularis	2	2.7
Pelvic inflammatory disease (PID)	2	2.7
Tubilovillous polyp	1	1.4
Meckel’s diverticulum	1	1.4
Carcinoid variant ^†^	7	1.7
Appendiceal adenocarcinoma ^‡^	1	0.2

Complications

The overall complication rate was 122 (29.2%), with the majority being in the laparoscopic group at 111 (26.6%). While most of the complications were observed in the minimally invasive group, this did not reach statistical significance (p=0.939). The most frequent morbidity reported was poorly controlled pain that led to a delay in hospital discharge, occurring in 33 (7.9%) patients, the majority in the laparoscopic group (p=0.755). The second most common morbidity reported was prolonged paralytic ileus, affecting 24 (5.7%) patients, with most cases occurring in the laparoscopic group (p=0.460); however, this was not statistically significant. The conservative management failure rate was 7 (1.7%). Most complications were in the Clavien-Dindo low categories, 58 (13.9%), and were predominantly in the minimally invasive group (p=0.818). Similarly, low complication rates were reported for superficial SSI, wound dehiscence, intestinal obstruction, and retained fecolith. Other reported postoperative outcomes were a 30-day representation rate of 24 (5.7%) and a readmission rate of 10 (2.4%) (Table 5). A further breakdown of the reasons contributing to the 30-day representation can be found in (Table 6), demonstrating that the development of phlegmon/collection accounted for most representations at 15 (62.5%).

**Table 5:** Summary of postoperative complications - combined (inpatient and outpatient). Values are the number (n) of participants and percentages (%) unless otherwise indicated. * Pearson Chi-Square analysis and Fisher’s Exact Test (for cell values <5), and * Bolded denotes significance at p<0.05. ^†^ Delayed - being representation beyond the initial 30-day follow-up.

Variables (N = 418)		Total n (%)	Laparoscopic n (%)	Open n (%)	P-value*
Postoperative outcomes	Any 30-day complication	122 (29.2)	111 (26.6)	11 (2.6)	0.939
	Pain delaying discharge	33 (7.9)	31 (7.4)	2 (0.5)	0.755
	Paralytic ileus	24 (5.7)	21 (5.0)	3 (0.7)	0.460
	Conversion to open	19 (4.5)	19 (4.5)	-	1.000
	Post-op deep-seated collection	16 (3.8)	16 (3.8)	-	0.381
	Urinary retention	15 (3.6)	13 (3.1)	2 (0.5)	0.633
	Delayed theatre – failed non-operative ^†^	7 (1.7)	6 (1.4)	1 (0.3)	0.480
	Surgical site infection	5 (1.2)	3 (0.7)	2 (0.5)	0.311
	Bowel injury	5 (1.2)	5 (1.2)	0 (0.0)	1.000
	Unplanned return to theatre	3 (0.7)	3 (0.7)	0 (0.0)	1.000
	Surgical site wound dehiscence	2 (0.5)	1 (0.2)	1 (0.2)	0.169
	Intestinal obstruction	2 (0.5)	2 (0.5)	0 (0.0)	1.000
	Retained faecolith	1 (0.2)	1 (0.2)	0 (0.0)	1.000
	Incisional hernia	1 (0.2)	1 (0.2)	0 (0.0)	1.000
	30-day representation	24 (5.7)	22 (5.2)	2 (0.5)	0.641
	30-day readmission	10 (2.4)	8 (1.9)	2 (0.5)	0.927
	Any cause mortality	0 (0.0)	0 (0.0)	0 (0.0)	-
	Clavien-Dindo Classification				
	I	58 (13.9)	51 (12.2)	7 (1.7)	0.818
	II	54 (12.9)	51 (12.2)	3 (0.7)	0.757
	III	10 (2.4)	9 (2.1)	1 (0.3)	0.852

**Table 6:** 30-day representation. Values are the number (n) of participants and percentages (%) unless otherwise indicated. ^†^ Calculated as a proportion of the sub-cohort.

Variables (n =24)	(n)	(%)
Phlegmon/collection	15	62.5%
Delayed post-op ileus	5	20.8%
Wound infection	4	16.7%
Failed conservative management	2	8.3%
Small bowel obstruction	2	8.3%
Wound dehiscence	2	8.3%

A further breakdown of the reasons contributing to the 30-day representation can be found in (Table 6), demonstrating that the development of phlegmon/collection accounted for most representations at 62.5%.

## Discussion

Clinical assessment remains the cornerstone of diagnosing AA. Longstanding controversy surrounds diagnostic imaging in AA, where the associated costs and perceived delay in treatment must be balanced against diagnostic benefits [7]. This study showed that clinical diagnosis was non-inferior in accuracy to radiological imaging technologies.

In this study, there was a heavy reliance on imaging to aid diagnosis, with almost 75% of the patients worked up for acute appendicitis undergoing imaging, and 10% had multiple imaging modalities. Younger patients were less likely to undergo imaging preoperatively, presumably because the likelihood of appendicitis as a clinical diagnosis is higher in this group. If they were to be imaged, it was in the form of USS. The most common imaging modality, unsurprisingly, was USS in the younger group. Older adults were more likely to have a CT, a trend previously reported in the literature. CT was more likely to pick up significant differential pathology in adults [7]. There was a considerable difference in the modality of imaging used, with CT being the most utilized (in the older age group), a finding consistent with the literature [8, 12, 15]. This study’s cohort's mean age was older than previous literature reports [8]; this could have influenced how the imaging studies were protocoled by the radiology department, leading to the predominance of CT. It is, however, essential to acknowledge that CT scans come with inherent risks, including radiation exposure, especially concerning younger female patients [16]. While we did not collect data on which team (ED or surgery) requested CT, we have noted a large number of CT scans being requested by ED without recourse to surgery (work in progress). Other considerations include potential delays in surgery and the financial costs associated with CT [15-17]. Consequently, several risk mitigation strategies and protocols have been developed to address these concerns.

This study highlights that while CT remains the predominant imaging modality for diagnosing AA, clinical diagnosis demonstrates comparable accuracy (79.2% for clinical compared to 82.4% for USS). As modern medicine progresses with increasing reliance on CT imaging, particularly with contrast-enhanced CT scans, it is essential to reconsider the role of non-contrast CT scans in future diagnostic approaches. Wang K et al. confirmed during the recent COVID-19 pandemic-driven global shortage of contrast that non-contrast CT scans were non-inferior to contrast-enhanced CT scans in diagnosing acute abdomen, including acute appendicitis [18]. A significant advantage of non-contrast CT is its ability to minimize ionizing radiation exposure.

There remains, however, a subgroup of patients that may benefit from CT following USS. This study demonstrated that equivocal USS has the second-highest NAR at 43.3%. More importantly, the NAR reduced significantly (27.3%) for the equivocal USS group that underwent a subsequent CT. Furthermore, this figure dramatically decreased to 6.7% if the CT had confirmed AA. When used cautiously, follow-up imaging could reduce patients undergoing unnecessary surgery and associated complications. Clinical selection of patients requiring further imaging is perhaps one of the most vital skills for surgeons to learn.

Pre-operative imaging has historically been associated with delays to surgery [19]. However, in this study, no significant statistical difference was found between having imaging, the modality of imaging, the number of imaging scans performed, and the average time to surgery. Of note is that the imaging group had a longer procedure duration and length of hospital stay than the no-imaging group, indicating more significant pathology than the imaging effect. This type of study was neither designed nor powered to investigate this assumption. The WSES guidelines have weighed in on the topic, albeit in a qualified statement limited only to uncomplicated presentations, and suggested that a surgical delay of up to 24 hours is safe in uncomplicated acute appendicitis and does not increase the risk of complications, perforation rate, and morbidity in adults [7]. The prolonged theater time reported in this study is also likely an inaccurate endpoint due to the varied experience of the surgeons performing the procedure, with a significant number of procedures done by trainees.

This study yielded unexpected findings, demonstrating that junior trainees exhibited a notably higher accuracy in intra-operatively assessing a macroscopically normal appendix than their more senior counterparts. This phenomenon may be attributed to the constant presence of a senior supervisor during surgery, potentially influencing the accuracy of intra-operative observations by having more personnel present in the theatre. This study found that a surgeon’s ability to judge appendicitis accurately intra-operatively was moderate, supporting the WSES recommendation to remove a normal-looking appendix intra-operatively if no other source of symptoms is identified [7]. This study’s findings of moderate intraoperative agreement on acute appendicitis were comparable to other international studies in New Zealand [20] and the United Kingdom [21].

Healthcare cost analyses are inherently complex. This study estimated direct diagnostic imaging costs at over $200,000 over 12 months in a single center, whereby 27% of scans yielded equivocal results. Although CT scans are almost four times the cost of USS scans, improved accuracy can mitigate the necessity for additional imaging in cases involving equivocal findings or the potential for a negative appendicectomy. Negative appendicectomies are frequent and result in an extra financial burden to the health service. D’Souza N et al. found that routine imaging for all patients with suspected appendicitis is cost-effective by reducing the NAR [22]. Alternatively, the WSES guidelines supported a staged imaging protocol to rationalize the cost associated with diagnostic scans by doing a USS first [7], where negative or equivocal USS results are followed by a CT. However, in this study, there was no statistically significant difference in the NAR of clinical 20.8% vs 17.6% in USS-reported appendicitis, negating the need for universal USS but rather a more targeted approach.

The retrospective design of this study presents a limitation in assessing factors influencing imaging outcomes. During the study period, only gender and age demographics were routinely recorded, with the absence of data on potential confounding variables. For instance, records of BMI were not accessible, which is noteworthy because it is widely acknowledged that USS scans have limited utility in individuals with elevated BMIs (≥25 kg/m^2) due to their higher rate of inconclusive results. Another limitation of this study is that it did not evaluate how the radiology department protocoled the scans. This study also had limited recording and varied usage of scoring systems pre-operatively, which limited their utility in analysis.

## Conclusions

This study showed that clinical diagnosis matches the diagnostic accuracy of imaging technologies. Utilizing diagnostic imaging methods promptly and appropriately did not lead to considerable delays in surgery. Surgeons' capability to diagnose appendicitis during surgery is moderately accurate. Most patients underwent imaging, with CT scans being the most common. Moving forward, practitioners must minimize excessive reliance on imaging techniques as this can be resource-intensive, especially in developing countries. Future clinical practice should balance embracing technological advancements and preserving essential clinical diagnostic expertise, as medicine is both a science and an art.
